# A cross-sectional study in healthy elderly subjects aimed at development of an algorithm to increase identification of Alzheimer pathology for the purpose of clinical trial participation

**DOI:** 10.1186/s13195-021-00874-9

**Published:** 2021-07-17

**Authors:** Samantha Prins, Ahnjili Zhuparris, Ellen P. Hart, Robert-Jan Doll, Geert Jan Groeneveld

**Affiliations:** 1 Centre for Human drug Research, Leiden, the Netherlands; 2 Leiden University Medical Center, Leiden, the Netherlands

**Keywords:** Alzheimer, Preclinical AD, Clinical trial, Algorithm, CSF Aβ

## Abstract

**Background:**

In the current study, we aimed to develop an algorithm based on biomarkers obtained through non- or minimally invasive procedures to identify healthy elderly subjects who have an increased risk of abnormal cerebrospinal fluid (CSF) amyloid beta42 (Aβ) levels consistent with the presence of Alzheimer’s disease (AD) pathology. The use of the algorithm may help to identify subjects with preclinical AD who are eligible for potential participation in trials with disease modifying compounds being developed for AD. Due to this pre-selection, fewer lumbar punctures will be needed, decreasing overall burden for study subjects and costs.

**Methods:**

Healthy elderly subjects (*n* = 200; age 65–70 (*N* = 100) and age > 70 (*N* = 100)) with an MMSE > 24 were recruited. An automated central nervous system test battery was used for cognitive profiling. CSF Aβ1-42 concentrations, plasma Aβ1-40, Aβ1-42, neurofilament light, and total Tau concentrations were measured. Aβ1-42/1-40 ratio was calculated for plasma. The neuroinflammation biomarker YKL-40 and APOE ε4 status were determined in plasma. Different mathematical models were evaluated on their sensitivity, specificity, and positive predictive value. A logistic regression algorithm described the data best. Data were analyzed using a 5-fold cross validation logistic regression classifier.

**Results:**

Two hundred healthy elderly subjects were enrolled in this study. Data of 154 subjects were used for the per protocol analysis. The average age of the 154 subjects was 72.1 (65–86) years. Twenty-four (27.3%) were Aβ positive for AD (age 65–83). The results of the logistic regression classifier showed that predictive features for Aβ positivity/negativity in CSF consist of sex, 7 CNS tests, and 1 plasma-based assay. The model achieved a sensitivity of 70.82% (± 4.35) and a specificity of 89.25% (± 4.35) with respect to identifying abnormal CSF in healthy elderly subjects. The receiver operating characteristic curve showed an AUC of 65% (± 0.10).

**Conclusion:**

This algorithm would allow for a 70% reduction of lumbar punctures needed to identify subjects with abnormal CSF Aβ levels consistent with AD. The use of this algorithm can be expected to lower overall subject burden and costs of identifying subjects with preclinical AD and therefore of total study costs.

**Trial registration:**

ISRCTN.org identifier: ISRCTN79036545 (retrospectively registered).

**Supplementary Information:**

The online version contains supplementary material available at 10.1186/s13195-021-00874-9.

## Background

As new disease-modifying therapies for Alzheimer’s disease (AD) enter clinical trials, identifying the disease at a clinical stage where the pathological injury is not too severe to allow functionally meaningful recovery, or at least stabilization, is a major issue of current research [1]. Classification criteria aim at defining early clinical, biochemical, and metabolic markers of AD before the clinical criteria of dementia are fulfilled [2]. Identification of the pre-dementia phase of AD is crucial to allow progress of new treatments designed to intervene in the disease process at the earliest possible stage.

The current leading hypothesis regarding the pathophysiology of AD is centered on the misfolding and aggregation of toxic amyloid beta (Aβ) species such as Aβ1-42, and drug research has therefore so far focused most on this therapeutic target. Emerging data in otherwise healthy elderly individuals suggest that biomarker evidence of Aβ accumulation and neurofibrillary tangles are associated with functional and structural brain alterations, consistent with the patterns of abnormality seen in patients with mild cognitive impairment (MCI) and even AD, prior to the clinical expression of symptoms [3]. A phase one study in 2016 showed promising results of the anti-Aβ antibody aducanumab in patients with prodromal and mild AD by decreasing Aβ plaques in the brain [4]. Following this phase one study, the compound was further studied in two identically designed phase 3 trials. In March 2019, the trial was halted due to ineffectiveness. Further analyses showed that in one of the two phase 3 trials the patient group that received the highest dose of the active compound showed slower cognitive decline than the placebo group [5]. Based on these results, the FDA recently approved aducanumab for the treatment of AD in the USA under the “accelerated approval pathway” which provides patients access to drugs when there is an expectation of clinical benefit despite some uncertainty about the clinical benefit of the drug [6]. Aβ immunotherapy could prevent (progression to) AD in healthy elderly who show evidence of amyloid pathology and could prevent (further) aggregation of neurotoxic forms of Aβ and would thereby prevent downstream effects as synaptic dysfunction, neuronal damage, and cognitive impairment [7]. However, many phase 3 anti-amyloid trials have failed to demonstrate effects on progression of cognitive decline in patients with (mild to moderate) AD, despite clear Aβ lowering effects in cerebrospinal fluid (CSF) or PET [8–12].

Based on extensive longitudinal biomarker studies [13, 14], a specific pattern of deterioration of AD specific biomarkers has been proposed, which reflects the underlying progressive neuropathology of the disease. In this model, described by Jack et al., [14] concentrations of Aβ in CSF start decreasing decades before clinical symptoms appear. Changes in total and phosphorylated tau (t-Tau, p-Tau) concentrations in CSF have been shown to occur up to 15 years prior to the clinical onset of AD [15, 16]. Studies with Aβ lowering compounds are increasingly performed in cognitively healthy subjects with a CSF profile consistent with AD or “preclinical AD,” due to this early decrease of Aβ in CSF and the hypothesis that cognitive deterioration can still be prevented at this stage [17, 18]. Over the age of 65, approximately 20% of cognitively healthy subjects can be expected to have a CSF profile with lowered Aβ levels consistent with AD as this is shown to be an age-related process [19]. This means that to identify a single healthy elderly subject with CSF values consistent with AD, four subjects will have to undergo a lumbar puncture unnecessarily. This leads to unnecessary overall burden for study subjects and to higher study costs.

In the clinical setting, the diagnosis of (probable) AD is made based on clinical symptoms (e.g., self-reported memory loss, partner reports, difficulties in daily functioning), combined with neuropsychological testing, and confirmed by evidence of amyloid pathology in CSF (abnormal Aβ and/or Tau levels) or on amyloid PET scans, when available. The collection of CSF is, however, an invasive technique, which is burdensome in itself but also carries a risk of adverse effects (e.g., post-puncture headache) while PET scans are time consuming, not available for all patients, and expensive [19, 20].

As a result of the aforementioned, many studies have attempted to identify blood assays which can reliably measure AD-related biomarkers [21, 22]. Some seem to be successful in making a distinction between blood Aβ levels in subjects with (subjective) cognitive impairment, MCI, or AD [23, 24]. Also, the biomarkers t-Tau and neurofilament light (NfL) have been able to make this distinction [25, 26]. Limitations of the current blood-based biomarkers are that outcomes are not consistent between studies and the methods used are highly diverse [27].

In the current study, we aimed to develop an algorithm based on minimally invasive biomarkers (plasma and cognitive tests), to be used for pre-selection of subjects with an increased risk of lowered, abnormal, CSF Aβ levels (“Aβ positive subjects”) consistent with the presence of AD pathology. This algorithm can be used to preselect cognitively healthy Aβ positive people for drug studies in preclinical AD, thereby resulting in fewer subjects needing to undergo a lumbar puncture.

## Methods

This was a single-center, cross-sectional, observational, correlational study. All study participants visited the research unit twice, once for a medical screening and once for the study measurements.

The study was approved by the ethics committee of the Leiden University Medical Center (LUMC), the Netherlands. The study was conducted according to the Dutch act on Medical Research Involving Human Subjects (WMO) and in compliance with Good Clinical Practice (ICH-GCP) and the Declaration of Helsinki. The trial was retrospectively registered in the international trial register with ID number: ISRCTN79036545.

### Participants

We aimed to enroll 200 healthy male and female participants, with an age of 65 years and older. Of these 200 subjects, at least 100 participants were to be above the age of 70. All the subjects visited Centre for Human Drug Research (CHDR) between October 2017 and November 2018 where all study assessments took place. CHDR is a clinical pharmacology research facility where early phase clinical drug studies and methodology and biomarker research are performed. For this study, a population of healthy elderly subjects aged 65 years and over was chosen as the prevalence of neurodegenerative disorders with an important cognitive component such as AD increases significantly from this age onwards [19]. Main exclusion criteria were a diagnosis of a cognitive disorder (including but not limited to MCI, AD, Lewy body dementia, frontotemporal dementia), history of psychiatric disease in the past 3 years, Mini Mental State Examination (MMSE) ≤ 24, Geriatric Depression Scale (GDS) ≥ 6, presence of drug or alcohol abuse (< 2 standard drinks per day for female and < 3 standard drinks per day for male), and any medication which influences the central nervous system or is contraindicative for the performance of a lumbar puncture.

All subjects underwent medical screening, including medical history, physical examination, vital signs measurements in supine and standing position, routine hematology, urinalysis, and urine drug screen.

All subjects visited the clinical research unit once for the study day and underwent blood sampling at predefined time points (0, 2, and 4 hour[s]). A single lumbar puncture was performed for the collection of CSF (at 4 h). Furthermore, a CNS test battery was performed to collect data on different CNS domains.

### Blood sampling

Approximately 10 mL blood was collected via an i.v. catheter placed in an antecubital vein in the arm in appropriate K2EDTA tubes at the predefined time points mentioned above. Immediately following collection of the required blood volume, the tubes were slowly tilted backwards and forwards (no shaking) to bring the anticoagulant into solution. The blood plasma samples for bioanalysis were centrifuged within 1 h, at 2000*g* for 10 min at 4 °C. Prior to centrifugation, plasma samples were kept at room temperature. Immediately after centrifugation, supernatant was divided into 0.5 ml aliquots in Sarstedt polypropylene 0.5 mL tubes and stored at − 80 °C.

### Lumbar puncture

A CSF sample of 4 mL was collected in a 10 mL polypropylene tube. CSF was centrifuged within 1 h, at 2000*g* for 10 min at 4 °C. Prior to centrifugation, CSF samples were kept at room temperature. Immediately after centrifugation, samples were divided into 0.5 ml aliquots in Sarstedt polypropylene 0.5 mL tubes and stored at − 80 °C. Lumbar punctures were performed by a trained, physician with a 25G atraumatic lumbar puncture needle (Braun, 25G) under supervision of an experienced neurologist. The needle was placed at the L3-L4 or L4-L5 interspace with the subject in supine or sitting position. If a subject suffered from post-dural headaches, the subject was treated according to our standard operating procedures.

### Amyloid status

Amyloid beta1-42 was measured in the CSF using the fully automated Elecsys platform as this is widely used for diagnostics [28]. Lowered Aβ levels classified as amyloid abnormal and consistent with the presence of Alzheimer pathology were dichotomized by creating a group of “Aβ positive subjects” (Aβ < 1000 pg/ml) and “Aβ negative subjects” (Aβ ≥ 1000 pg/ml).

### Plasma analysis

Several plasma analyses were performed in plasma samples that were taken within 1 h from the CSF sample. Plasma biomarkers have been selected based on promising previous research of the use of plasma biomarkers to predict AD pathology. Although analytical methods vary, previous research has been able to measure Aβ, t-tau and NfL in plasma and have therefore been included to this study and the algorithm [23–26]. Plasma concentration of Aβ 1-40, Aβ 1-42, t-Tau, and NfL were measured using the fully automated, highly sensitive single molecule array Simoa technology [29]. The Aβ scores have been used as single variables as well as in a ratio score Aβ 1-42/Aβ 1-40.

Chitinase 3-like 1 (CHI3L1), or more commonly called YKL-40, is a glycoprotein which is mainly expressed in astrocytes. Insoluble Aβ aggregates in the brain can induce the activation of microglia, resulting in the synthesis of proinflammatory mediators, which further can stimulate astrocytic expression of YKL-40 [30]. Higher concentrations of YKL-40 were found in patients with prodromal AD, MCI, and full-blown AD [31, 32] when measured in CSF. Measuring YKL-40 in plasma can lead to a less invasive method of measuring inflammation related to AD in healthy subjects. YKL-40 was measured in plasma samples using the CHI3L1 Human ELISA Kit (Thermo Fisher).

### Apolipoprotein E genotyping

Apolipoprotein E (APOE) genotyping was performed after isolating DNA from EDTA blood. DNA was isolated using QIAamp DNA Blood MINI kit after which a polymerase chain reaction (PCR) technique was applied on the clean DNA. A sequential analysis (according to the Sanger method) than determined the APOE genotype. One or 2 APOE ε4 alleles classified subjects as APOE ε4 carriers, when no APOE ε4 alleles were present a subject was classified as noncarrier.

### Cognitive assessments and questionnaires

The NeuroCart is a battery of CNS tests used to assess a wide range of CNS domains [33]. All measurements were performed in a quiet room with ambient illumination. Per session, there was only one participant in the room. The following tests were performed using the NeuroCart: the Adaptive tracking test to measure attention and eye-hand coordination [34], the Face encoding and Recognition task (FACE) to measure visual memory [35], the Visual Verbal Learning Test (VVLT, 30 words) to measure the whole scope of learning behavior (i.e., acquisition, consolidation, storage and retrieval) [36], the Milner Maze test (MMT) to evaluate visuospatial working memory [37], the N-Back test to evaluate working memory [38], the Sustained Attention to Response Task (SART) as a vigilance task [39], and finger tapping for motor fluency [40], and saccadic and smooth eye movement [41] were also measured.

21-Leads electro encephalography (EEG) [42] recordings were made for all subjects to monitor (abnormal) brain activity. An 8-min resting EEG was performed while the subjects alternated 4 min with their eyes closed and 4 min with their eyes opened while resting in a chair. Subjects face a featureless wall and are instructed not to stare, not to move their head and eyes, and to suppress eye blinks. The Refa-40 (TMSi B.V., the Netherlands) recording system and 32-lead cap (TMSi B.V.) have been used. The five standard EEG band have been analyzed: delta (1.5 < 6.0), theta (6.0 < 8.5), alpha (8.5 < 12.5), beta (12.5 < 30.), and gamma (30.0 < 40.0).

The clinical dementia rating scale (CDR) [43] was assessed via a semi-structured interview with the participating subject only, to rate impairment in six different cognitive categories (memory, orientation, judgment and problem solving, community affairs, home and hobbies, and personal care). To rate impairment in more complex daily activities, the Instrumental Activities of Daily Living Scale (IADL) [44] was assessed. Both questionnaires were administered by trained neuropsychologists.

### Sample size justification

In this study, we selected elderly at the age of 65 years old and higher of which at least a hundred above the age of 70. According to Jansen et al. [19], we expected at least 19% amyloid pathology in a 65+ population and 23% amyloid pathology among cognitively healthy 70+ elderly subjects. We expected more responsiveness for study participation from elderly between the age of 65 and 70, based on our experience with previous studies with participants in this age range. Participants in this age range have participated in studies at CHDR before and are therefore registered in our local database and have received emails about this study. A higher number of participants within the age range 65–70 are present in the database compared to older elderly. Therefore, we aimed to enroll at least 100 subjects of > 70 years old in this study as prevalence of amyloid pathology is expected to be higher in this age group. This would result in an estimated 23 Aβ positive subjects versus approximately 77 Aβ negative subjects in the > 70 years old age group. Along with approximately 19 Aβ positive subjects versus 81 Aβ negative subjects in the age group 65–70, we expected to identify at least 42 Aβ positive healthy elderly subjects among the total group of 200. Based on previous comparable studies, these numbers were considered appropriate for a correlational study aimed at defining an algorithm [45, 46].

### Statistical analysis

Statistical analyses were performed using Python (version 3.7.3) and the sklearn package (version 0.21.3). To build a classification model that could differentiate between Aβ positive subjects and Aβ negative subjects, all parameters such as plasma data, genetic information, cognitive assessments, level of education, age, and gender were included as features.

When a classifier contains more features than can be justified by the observed data, there is a risk of the model overfitting. Overfitting occurs when a classifier corresponds too closely to a particular subpopulation and cannot be generalized to the wider population. Two methods were used to reduce the feature space, variance inflation factor (VIF) and penalized regression. VIF identified the pairs of highly correlated features and subsequently removes one of the features from the classifier. Penalized regression was applied to the logistic regression classifier to shrink the coefficients of features that were less predictive of the outcome.

For this study, we reviewed the performance of four classifiers—ridge-penalized logistic regression, random forest classifier, support vector machine classifier, and k-nearest neighbors classifier—on four datasets—a dataset with all features, only the VIF-selected features, all features except the EEG features, and all features except the genotyping feature. To ensure that the models were not under- or overfitting, we performed 5-fold stratified cross-validation. This data partitioning approach ensures that we built a more generalized model that can perform well when presented with unseen data. The 5-fold stratified cross-validation randomly samples the data into 5 folds of approximately equal proportions. In this case, there were 30 or 31 subjects per fold. Each fold contained the same ratio of Aβ positive and Aβ negative subjects. The model was trained on 4 folds of data and validated on the 5th fold. The cross-validation process was repeated 5 times, with each of the subsamples used exactly once as the validation data. The validation results were averaged over each iteration to estimate the model's predictive performance. We selected the optimal classifier by selecting the classifier with the highest sensitivity and specificity. If the sensitivity and specificity scores were identical between classifier, we then choose the classifier with the highest F1 score.

## Results

### Demographic and clinical characteristics

Two hundred healthy elderly subjects were enrolled in this study of which 189 were included in the CSF and plasma analyses due to CSF availability. The 11 missing CSF samples were due to absent CSF flow during lumbar puncture. The 189 CSF samples were analyzed on Aβ42 using the Elecsys method and 55 healthy elderly had CSF Aβ42 levels consistent with AD (Aβ < 1000 pg/ml). Of the 189 subjects with CSF availability, 154 subjects were included in the per protocol analyses. Plasma analyses were missing for 27 subjects due to analytic errors. NeuroCart data was incomplete for 8 subjects. Forty-nine subject were female (68.2% were male and 31.8% female). Their mean age was 72.1 years (range 65–86), with a median MMSE score of 29 (range 25–30), and GDS score of 0 (median, range 0–5). Self-reported memory performance and daily functioning were assessed using CDR and IADL scores with averaged scores of 0 in all subjects. Of the 154 elderly, 42 (27.3%) were Aβ positive for AD (average age 73.7 [65–83]; see Table 1).

**Table 1:** Demographics, clinical characteristics, and biomarker information of the study population

Characteristics	Total group, *n* = 154	Amyloid status CSF	
		Aβ positive, *n* = 42 (27.3%)	Aβ negative, *n* = 112 (72.7%)
Age, years	72.1 [65; 86]	73.7 [65; 85]	71.4 [65; 86]
Female gender	49 (31.8 %)	13 (30.6%)	36 (32.1%)
MMSE	29 (25–30)	29 (25–30)	29 (25–30)
GDS	0 (0–5)	1 (0–5)	0 (0–5)
CDR	0.0 (0–0.5)	0.0 (0)	0.0 (0–0.5)
IADL	0.0 (0)	0.0 (0)	0.0 (0)
Education^a^	6 (1–7)	6 (1–7)	6 (1–7)
Apoe e4/e4 (*n* = 150)	5 (3.3%)	5 (100%)	0 (0%)
Apoe at least one e4 allele (*n* = 150)	39 (26%)	18 (42.9%)	21 (18.8%)

Continuous data are presented as mean [min; max] and dichotomous data as n (%). MMSE, Mini Mental State Examination; GDS, Geriatric Depression Scale; CDR, Clinical Dementia rating Scale; IADL, Instrumental Activity of Daily Living scale; Apoe e4, apolipoprotein E 4.

^a^Level of education defined as (1) lower than primary school, (2) primary school, (3) less than lower professional education, (4) lower professional education, (5) mid-level professional education, (6) high school/college, and (7) university

### Data analysis

**Fig. 1:**
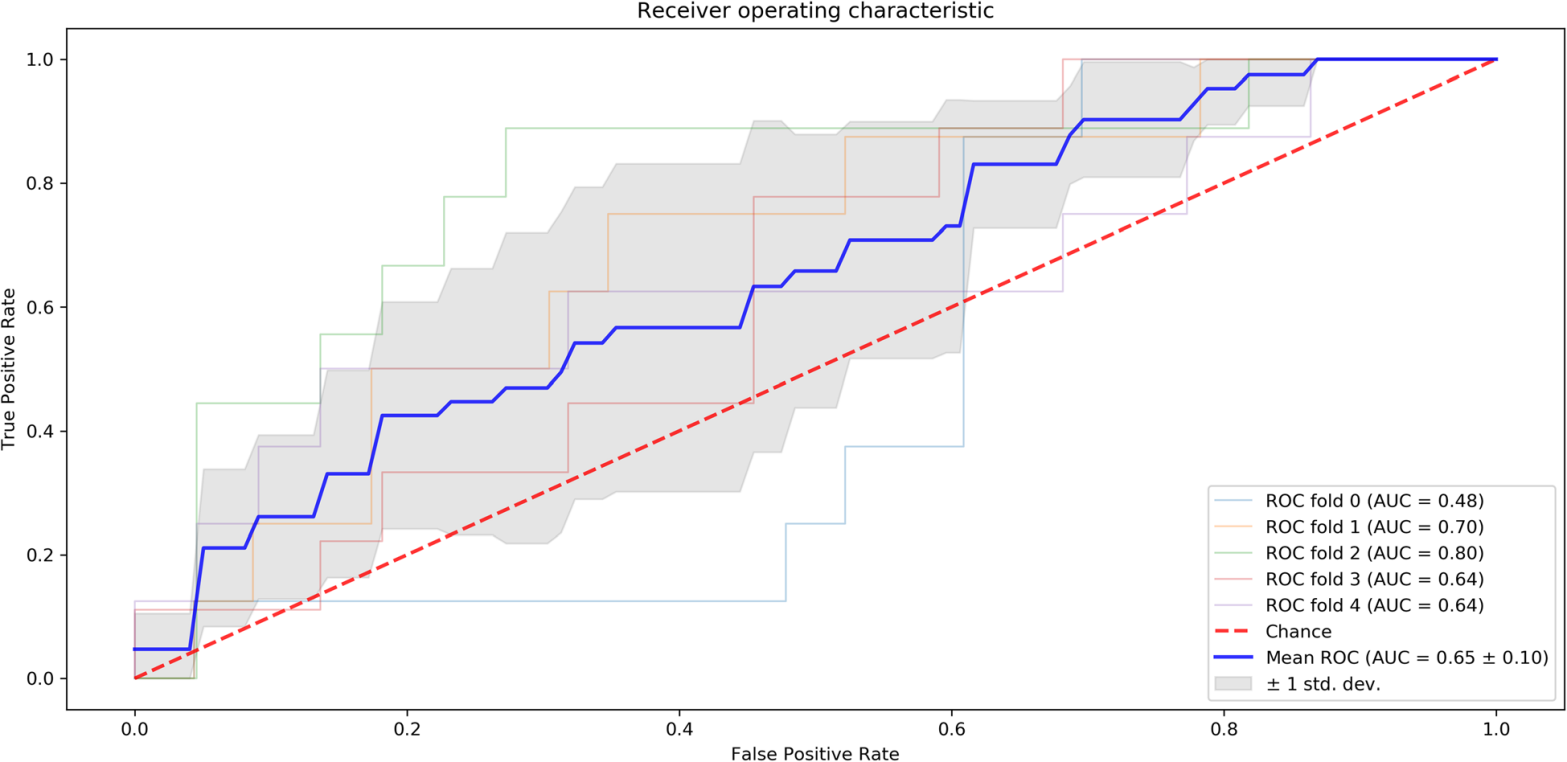
Receiver operating characteristic (ROC) metric to evaluate the logistic regression output quality using 5-fold cross-validation

For each dataset and classifier, we calculated the sensitivity, specificity, precision and F1 score. The VIF-selected features dataset and logistic regression classifier achieved a sensitivity and specificity of 70.8% and 89.2%. The receiver operating characteristic (ROC) curve showed an AUC of 65% (± 0.10); see Fig. 1. However, the mean performance for all four classifiers (for the VIF-selected features dataset) was 68.0% sensitivity and 76.4% specificity. The lowest sensitivity and specificity for the worse performing model (the random forest) was 63.6% and 70.6% respectively. While we found similar performance when applying different classifiers, the logistic regression showed the highest sensitivity and specificity for the classification task.

The best performing classifier, logistic regression, included 32 of the 90 parameters measured in this study. The results of the logistic regression algorithm analyses conclude that the best prediction of Aβ positivity/negativity in CSF in an elderly subject is made by combining the 32 parameters measured with the NeuroCart (Table 2). The algorithm included the following 7 CNS tests and 1 plasma analysis: MMT, VVLT, finger tapping, N-Back, SART, Face, EEG, and the plasma biomarker YKL-40. Sex was also included. We can use the logistic regression equation to calculate the probability (between 0 and 1) of a new subject being classified as amyloid positive or negative. If the subject is given a probability greater than 0.5, they will be classified as amyloid positive.

**Table 2:** NeuroCart activities and parameters included in the algorithm

Activity	Cognitive domain	Parameter
Visual Verbal Learning Test (VVLT, 30 words)	Memory	- Delayed word recall number correct - Immediate word recall number doubles, 3e trial - Immediate word recall number incorrect 1st trial - Delayed word recall number doubles - Immediate word recall number doubles, 2e trial - Immediate word recall number doubles, 1st trial - Immediate word recall number incorrect 3e trial - Delayed word recognition number incorrect - Immediate word recall number incorrect 2e trial
Electroencephalography (EEG)	Electrical brain activity	- Delta-power Fz-Cz (eyes open) - Theta-power Fz-Cz (eyes closed) - Beta-power Fz-Cz (eyes open) - Gamma-power Pz-O2 (eyes open) - Delta-power Pz-O2 (eyes open) - Gamma-power Pz-O1 (eyes closed) - Alpha-power Fz-Cz (eyes open) - Theta-power Pz-O1 (eyes open) - Gamma-power Fz-Cz (eyes open) - Alpha-power Pz-O1 (eyes closed)
Finger Tapping	Motor activation and fluency	- Standard deviation of the mean (dominant hand)
Sustained Attention to Response Task (SART)	Vigilance	- Total omission errors - Post error slowing
N-Back	Working memory	- Number correct—number incorrect/total for one back
Milner Maze test (MMT)	Spatial working memory	- Reversed total illegal moves - Immediate total repeat errors - Immediate total illegal moves - Delayed total illegal moves - Reversed total repeat errors - Delayed total repeat errors
Face encoding and recognition task (Face)	Episodic memory	- Number incorrect

Top activities/parameters have more impact on the algorithm than the bottom activities in this table

As EEG- and genotyping-based assessments are time and resource consuming tasks, we built two additional classification models excluding these features. By excluding the EEG features, the highest sensitivity and specificity achieved was 70.6% and 73.5%, respectively, using ridge-penalized logistic regression classifier. Hence, the exclusion of the EEG features had little to no effect on the sensitivity of the classifier but lead to a 15 percentage point drop in specificity compared to the best performing logistic regression model. When omitting the genotyping features (the APOE E4 status), the best performing model was the k-nearest neighbor. This model achieved a sensitivity and specificity of 70.4% and 72.3% respectively. Like the classifier with no EEG features, the exclusion of the genotyping data had little to no effect on the classifier’s sensitivity, while the specificity did drop by 16 percentage points compared to the best performing logistic regression model.

**Fig. 2:**
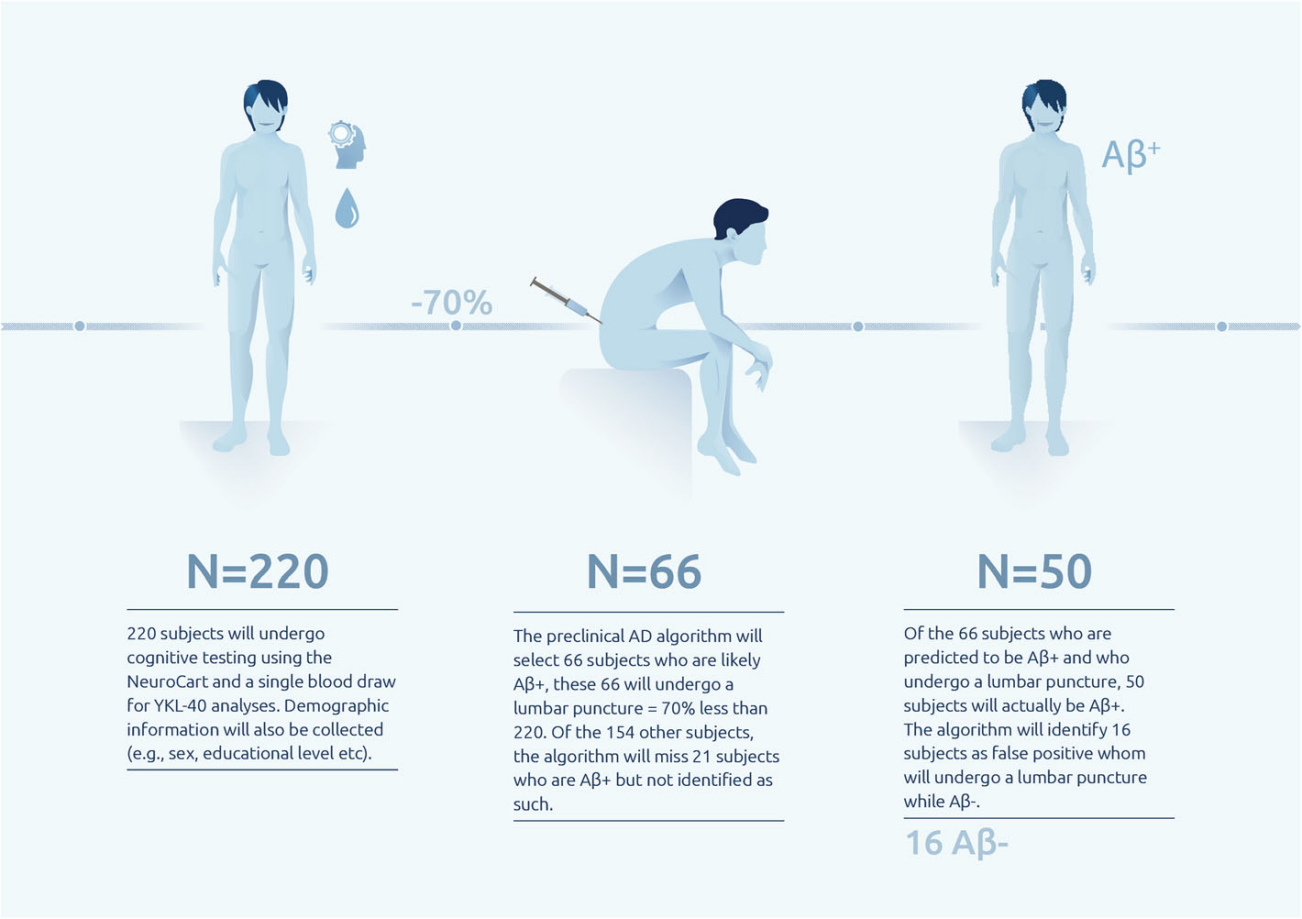
Visualization of reduction of lumbar punctures using the algorithm

When aiming for 50 healthy elderly with Aβ CSF levels consistent with AD, 220 elderly must undergo the (non-invasive) tests included in the algorithm. Of these 220 subjects, the algorithm will predict 66 elderly with Aβ positive levels in CSF, 50 of which will be true positives (Aβ CSF levels consistent with AD), the remaining 16 will be false positive (Aβ negative). However, 21 Aβ positive subjects will be mislabeled as Aβ negative (see Table 3). This algorithm would allow for a 70% reduction of lumbar punctures needed to identify subjects with abnormal CSF Aβ levels consistent with AD, meaning 66 lumbar punctures instead of 220 (see Fig. 2).

**Table 3:** Sensitivity/specificity table of the logistic regression algorithm

	Predicted Aβ +	Predicted Aβ -	Total
Actual Aβ +	50	21	71
Actual Aβ -	16	133	149
Total	66	154	220

Sensitivity and specificity table calculated with a sensitivity of 70.82% and specificity of 89.25%. When aiming for 50 positively predicted Aβ positive subjects, 66 will be predicted as such. Therefore, 16 subjects will falsely be predicted as being Aβ positive and 21 will falsely be predicted as being Aβ negative

## Discussion

This study aimed to develop an algorithm based on less-invasive (plasma) biomarkers for AD pathology, to be used for pre-selection of subjects who are suspected of lowered, abnormal, CSF Aβ levels (“Aβ positive subjects”) consistent with the presence of AD pathology. The algorithm includes sex, 7 cognitive tests measured with the NeuroCart (MMT, VVLT, finger tapping, N-Back, SART, Face, and EEG), and one plasma biomarker (YKL-40) and was successful in predicting CSF Aβ + in healthy elderly with a sensitivity of 70.82% and specificity of 89.25%. When using this algorithm, 70% fewer lumbar punctures will have to be performed to enroll subjects based on lowered Abeta CSF. The overall subject burden and costs of trials will reduce as fewer lumbar punctures will need to be performed. This may also increase subject’s willingness to participate.

Four classification algorithms (random forest, logistic regression, support vector machine classifier, and a K-nearest neighbors classifier) were used to classify Aβ positivity. A comparison of classification models is necessary to identify a model that best fits the data. Logistic regression outperformed the other algorithms in terms of accuracy, precision, and recall. The logistic regression model is ideal for Aβ positivity classification as it provides an estimation of the association between the predictor and the outcome. Palmqvist et al. [47] and Jang et al. [48] have also demonstrated the use of logistic regression to reliably dichotomize amyloid status using plasma. This further supports the notion that logistic regression can use multimodal non-invasive cognitive and blood-based biomarkers to stratified enrollment of subjects with preclinical AD into clinical trials. In this study, 200 healthy elderly were included of which 154 were eventually included in the model. This is a satisfactory amount of subjects to support the conclusion of this study. For the logistic regression classifier, we have selected 0.5 to be the probabilistic threshold for classifying a patient as Aβ + or Aβ−. Using the ROC curve (Fig. 1), a researcher may choose a different threshold depending on what they choose to prioritize, the true positive rate (sensitivity) or the false positive rate (1-specificity)).

Approximately 50 subjects is an acceptable number for a proof-Of-concept study of a novel compound; 20–80 subjects is common in phase one trials according to the FDA [49]. Based on the 27.3% Aβ positivity in our study, we estimate that in a new group of 220 healthy elderly, 71 subjects will be Aβ+. The algorithm will identify 66 subjects as having Aβ + CSF. Due to the sensitivity of 70.82%, 21 Aβ + subjects would not be identified as such. Also, 16 Aβ-subjects would wrongfully be identified as Aβ + which results in 50 truly Aβ + subjects. Using the algorithm would reduce the number of lumbar punctures in healthy elderly by 70%, i.e., 66 lumbar punctures instead of 220. As this algorithm is designed to select healthy elderly with Aβ CSF concentrations consistent with AD, having a 100% accuracy is of no importance, contrary to when using a test or an algorithm for diagnostic purposes. We would not perform unnecessary lumbar punctures in 89.25% patients with an increased chance of being Aβ-. In our opinion, this decrease in overall burden justifies the use of such an algorithm for subject selection for trial.

Other studies developed algorithms focused on predicting the progression to dementia due to AD [50, 51], the classification of different stages of AD [52, 53], and for the diagnosis of AD in the early stages [54]. These algorithms were developed for diagnostic purpose rather than for clinical trial participation, such as the one described in our study. Also, the data used in these algorithms were collected in clinical settings such as behavioral observation, clinical presentation, and MRI data. When selecting healthy elderly for clinical trial participation, this information is commonly not available. Others have tried to identify healthy subjects with amyloid pathology using considerably burdensome and costly MRI data [55, 56]. Khan et al. (2018) suggests an algorithm for preclinical diagnosis of AD based on a combination of three AD biomarkers: neuroimaging, genetic markers, and abnormalities in CSF Aβ1-42, t-tau, and p-tau (the gold standard for the diagnosis of AD). However, as mentioned before, data from neuroimaging is not commonly available and far more costly and time consuming than the tests used in our algorithm. Reduction of the number of lumbar punctures performed in healthy subjects is of great value to increase participation willingness in healthy elderly and to lower overall subject burden. A comparable study to this current study showed that Aβ positivity (confirmed by either CSF or PET-MRI) can be predicted by a combination of demographic variables, APOE status, baseline cognition, and 24-month follow-up rates [57]. A 24-month follow-up is usually not available and gathering follow up information on healthy subjects before the start of a clinical trial is too time consuming.

Accumulation of Aβ plaques in the brain associated with lowered levels of Aβ in CSF is still seen as the main pathological cause of AD. Various clinical trials have therefore focused on reducing Aβ plaques in the brain. Where reducing Aβ has been successful, lowering the prevalence of dementia due to AD has not been a result. Huang et al. (2020) reported 9 failed phase 3 anti-amyloid trails since 2016 with 6 different compounds [58]. Two of these trails were performed in subjects with preclinical AD, both with BACE inhibitors [17, 18] and both were discontinued due to either toxicity or lack of efficacy. Researchers claim that interfering early in the disease process will probably result in higher efficacy than when the clinical disease process has already started, evidenced by a diagnosis of preclinical AD or MCI. Looking at the inclusion criteria of the aforementioned studies shows that healthy elderly with CSF Aβ levels consistent with AD have been selected for participation. Healthy elderly are defined as having a clinical interview, namely the clinical dementia rating scale (CDR) of 0. Using the CDR total score is well accepted in clinical research and is widely used for clinical diagnosis of AD [59]. Still, very subtle cognitive changes are not detected using this crude screening tool. Using the algorithm proposed in this article will help to better select trial participants by including diverse cognitive assessments instead of the more general cognitive score of the CDR.

Shifting focus from invasive measurements (CSF, PET-MRI) to blood-based biomarkers for AD has been a major topic in research as new technics have been developed claiming to be ultrasensitive to detecting AD-related proteins [24]. Using a blood test would make it more accessible to diagnose patients but also to identify possible trial participants. Challenges in the use of blood-based AD biomarkers are the different biological system compared to the CSF system, use of different analytical methods (ELISA, Simoa, etc.), and the specificity for AD of these biomarkers [27]. Specifying pre-AD stages with the use of blood-based biomarkers has yet to be standardized. The preclinical AD algorithm created in this study includes only one blood-based biomarker (YKL-40) and the limitations of using blood-based biomarkers are therefore minor. The use of a different analytical method may alter the outcome of the analysis slightly and therefore could have led to a different composition of the algorithm. This should be kept in mind when comparing the outcome of this study to those of other studies. The combination of blood-based biomarkers with genetic information and cognitive assessments appears to be a powerful tool in preselection of preclinical AD subjects in clinical trials.

Four out of the seven NeuroCart tasks that are included in the algorithm are memory tasks. Loss of memory early on in the disease process is common for (amnestic) MCI and often lead to the AD diagnosis [60]. Especially the visual verbal learning task is important for the algorithm to differentiate between preclinical AD and healthy elderly. Visual and verbal memory problems are common in AD [61] and have also been reported in preclinical AD [62, 63].

Reducing the number of lumbar punctures in healthy subjects and the additional benefits for clinical research must be weighed against the ethical consequences of identifying healthy subjects with an elevated risk of developing AD, which at this moment is an untreatable disease. Approximately 53% of subjects fulfilling the criteria of preclinical AD will actually develop MCI or AD [64]. When selecting trial subjects based on specific biomarkers, these subjects will become aware that they have CSF Aβ levels consistent with AD. The development of Aβ plaques in the brain and eventually developing AD can be a 20- to 30-year-long process [19]. This is a substantial amount of time to be concerned about a disease that one might develop. Knowledge about predispositions to develop a disease can even have financial consequences and reduce health benefits as people might not be hired for certain jobs and health insurances may increase insurance premium. Nevertheless, studying cognitively healthy elderly is important as treatment in a pre-disease phase might prevent or retard the process of developing clinically overt Alzheimer’s dementia. With the ultimate goal of preventing AD, the need to include preclinical subjects in clinical studies is vital.

## Limitations

Among the limitations is that a logistic algorithm was used which cannot incorporate incomplete datasets [65]. Hence, the model will fail to predict a class if a subject is missing a single feature. Missing data is not uncommon in research, especially when cognitive tests are performed. Benefits of using this model however proceed this limitation. Inconclusiveness about the validity of blood based biomarkers can also be regarded as a limitation of this study. This study only includes one plasma biomarker which reduces the inconvenience. The ethical consequences of using an algorithm like ours in healthy elderly should always be taken into account and could be regarded as a limitation. The study population is a relatively highly educated group. This might not be completely representative with regards to the cognitive performance of an average population.

## Conclusion

This algorithm would allow for a 70% reduction of lumbar punctures needed to identify subjects with abnormal CSF Aβ levels consistent with AD. We have identified an algorithm that is able to preselect healthy elderly who are more likely to have Aβ CSF levels consistent with AD. Using this algorithm, fewer lumbar punctures will have to be performed when selecting subjects for clinical trials. The use of this algorithm can be expected to lower overall subject burden and costs of identifying subjects with preclinical AD and therefore of total study costs.

## Supplementary Information

**Additional file 1.** STARD flow chart.

## Data Availability

The datasets generated during this study are not available by request.

## Abbreviations

Aβ: Amyloid beta
Aβ+: Amyloid beta positive
AD: Alzheimer’s disease
Apoe: Apolipoprotein
AUC: Area under the curve
CHDR: Centre for Human Drug Research
CSF: Cerebrospinal fluid
CDR: Clinical dementia rating scale
EEG: Electro encephalography
IADL: Instrumental activities of daily living scale
MCI: Mild cognitive impairment
MMSE: Mini mental state examination
MMT: Milner Maze test
MRI: Magnetic reasoning image
NfL: Neurofilament light
SART: Sustained attention to response task
PET: Positron emission tomography
ROC: Receiver operating characteristics
VVLT: Visual verbal learning test
VIF: Variance inflation factor
