# Supplementary figures and images for: A cross-sectional study in healthy elderly subjects aimed at development of an algorithm to increase identification of Alzheimer pathology for the purpose of clinical trial participation
Source: Alzheimers Res Ther. 2021 Jul 17;13:132. doi: 10.1186/s13195-021-00874-9 (PMC8286577; doi:10.1186/s13195-021-00874-9)

# STARD flow chart

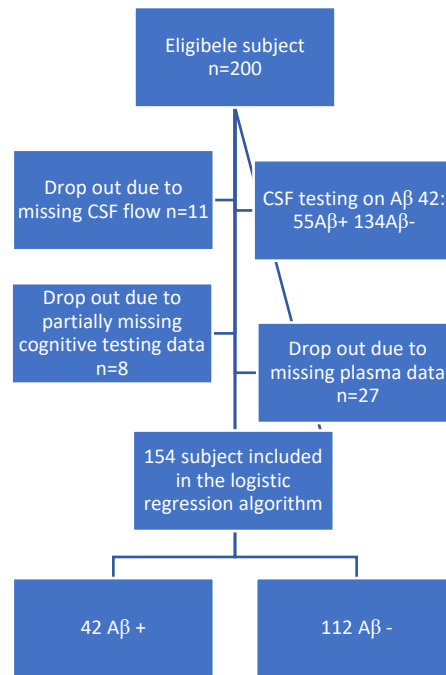

Supplement: Supplementary file 1 — Additional file 1. STARD flow chart. [file 13195_2021_874_MOESM1_ESM.pdf]
